# Maternal overweight and obesity and its associated factors and outcomes in human immunodeficiency virus (HIV)‐infected and HIV‐uninfected black South African pregnant women

**DOI:** 10.1111/jog.15392

**Published:** 2022-08-23

**Authors:** Christen R. Erasmus, Anil A. Chuturgoon, Niren R. Maharaj

**Affiliations:** ^1^ Department of Dietetics and Human Nutrition University of KwaZulu‐Natal Pietermaritzburg South Africa; ^2^ Department of Medical Biochemistry University of KwaZulu‐Natal Durban South Africa; ^3^ Department of Obstetrics and Gynaecology Prince Mshiyeni Memorial Hospital Durban South Africa

**Keywords:** human immune‐deficiency virus, hypertensive disorders, obesity, overweight, pregnancy

## Abstract

**Aim:**

This study aimed to investigate various variables between maternal overweight and/or obesity versus normal‐weight pregnant black South African women living with and without human immunodeficiency virus (HIV).

**Methods:**

A cross‐sectional study design was employed. A total of 200 pregnant women were enrolled in the study, categorized according to body mass index (BMI) (kg/m^2^) into two groups: (1) overweight/obese (≥25 kg/m^2^) (*n* = 97); and (2) nonoverweight/nonobese (<25 kg/m^2^) (*n* = 103), where 90 were HIV‐infected and 110 were HIV‐uninfected. The differences between the maternal BMI categories were assessed using Fisher's exact *t*‐test and the *χ* ^2^ test. Simple and multiple logistic regression analyses were used to determine factors associated with maternal overweight and obesity.

**Results:**

Multiple logistic regression analysis showed that maternal age (odds ratio [OR]: 1.061; 95% confidence interval [CI] 1.008–1.117; *p* = 0.023) and gestational age (OR: 1.121; 95% CI 1.005–1.251; *p* = 0.041) were significantly associated with maternal overweight/obesity in both HIV‐infected and HIV‐uninfected. For maternal health outcomes, multiple logistic regression analysis showed that hypertensive disorders (OR: 0.273; 95% CI 0.124–0.601; *p* = 0.001) and anemia (OR: 2.420; 95% CI 1.283–4.563; *p* = 0.006) were significantly associated with maternal overweight/obesity in both HIV‐infected and HIV‐uninfected. The overweight/obese HIV‐infected participants (OR: 0.233; 95% CI 0.075–0.717; *p* = 0.011) had increased odds for developing hypertensive disorders compared to HIV‐uninfected overweight/obese participants (OR: 0.471; 95% CI 0.172–1.291; *p* = 0.143).

**Conclusions:**

Maternal overweight/obesity in both HIV‐infected and HIV‐uninfected pregnant black South African women was significantly associated with maternal age, gestational age, HPT disorders, and anemia. Maternal overweight/obesity decreased the odds for anemia, but increased the odds for the development of HPT disorders, especially in the HIV‐infected pregnant women.

## Background

Maternal overweight and obesity in pregnancy have largely been investigated for its association with unfavorable clinical outcomes for both mother and child. With a focus on the mother, the health risks associated are dependent on the linearity of the body mass index (BMI) of the mother before, during, and after pregnancy.^1^ Hence, in terms of the risks for adverse maternal health outcomes during pregnancy these may include metabolic conditions like gestational diabetes mellitus (GDM), cardiovascular disease like hypertensive (HPT) disorders, as well as other complications like cesarean section (CS) birth, failed induction of labor, preterm rupture of membranes, venous thromboembolism, sepsis, and postpartum hemorrhage.^2^ Therefore, there is a need to better understand how to prevent overweight and obesity in pregnancy so that the risks for these adverse maternal health outcomes might be mitigated. In South Africa, up to 40% of pregnant women are living with human immunodeficiency virus (HIV) infection, and of those 30%–45% are classified as obese.^3^ Hence, population‐specific methodology should be applied to investigate the unique risk factors that exist to encourage overweight and obesity in pregnant women within a South African context, and these factors may include the diet, physical activity, lifestyle choices, and demographic characteristics.^4^, ^5^, ^6^ Therefore, this study sought to investigate various variables between maternal overweight and/or obesity versus normal‐weight pregnant black South African women living with and without HIV.

## METHODS

### Sample selection and study population

**FIGURE 1:**
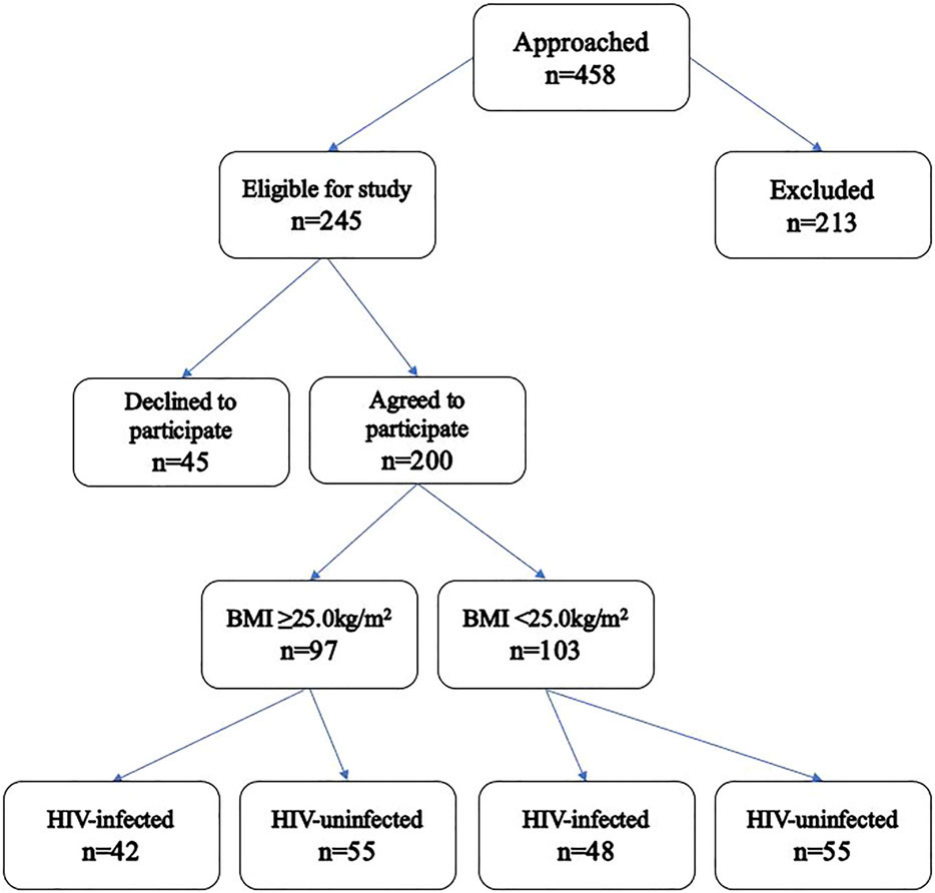
Eligibility flow diagram

A cross‐sectional study design was employed. Sample selection was conducted at Prince Mshiyeni Memorial Regional Hospital (PMMH), which is situated in Umlazi within the eThekweni municipality, KwaZulu‐Natal, South Africa. The catchment area for the hospital includes both rural and urban geographical areas. Pregnant women admitted to the labor ward were approached to participate in this study. The inclusion criteria for this study were as follows: (1) ≥18 years of age; (2) pregnant females; (3) Black South African citizen; (4) clinically stable; (5) able to stand without assistance; and (6) given verbal and written consent to participate in the study. The participants were categorized according to BMI (kg/m^2^) into two groups: (1) overweight/obese pregnant women (≥25 kg/m^2^); and (2) nonoverweight/nonobese pregnant women (<25 kg/m^2^). A total of 458 pregnant women were approached to participate in the study, but 245 subjects met the inclusion criteria and of these 45 declined to participate. Hence, a total of 200 subjects met all the inclusion criteria. The 200 pregnant women were categorized into BMI ≥25 kg/m^2^ (*n* = 97) and BMI <25 kg/m^2^ (*n* = 103), where 90 were HIV‐infected on antiretroviral treatment (ART) and 110 were HIV‐uninfected (see Figure 1).

### Maternal anthropometric assessment

Anthropometric measurements were conducted by an international society for the advancement of kinanthropometry level 1 trained dietician. In order to avoid anthropometric measurement errors, all measurements were conducted by the same researcher, taken three times, and recorded to the nearest 0.1 cm/kg.^7^, ^8^ The mean of the two closest values was recorded.^7^, ^8^ The standing height (SH) measurement was measured via. Stretch stature methodology using a portable calibrated stadiometer (Seca) with a sliding headboard.^9^ The weight measurement was taken using actual body weight (ABW) methodology, using a portable calibrated scale (Seca, with a maximum weight threshold of 250 kg).^10^ The scale was calibrated before the commencement of the study. BMI was calculated using the weight of the mother (MW) post‐delivery. The BMI was interpreted according to the BMI (MW/SH^2^ = kg/m^2^) classifications: (1) overweight/obese pregnant women (≥25 kg/m^2^); and (2) nonoverweight/nonobese pregnant women (<25 kg/m^2^).^11^

### Maternal interviews

A trained dietician conducted a one‐on‐one interview with each participant using an adapted version of a validated questionnaire that was made available in English and isiZulu (an indigenous South African language).^12^ The isiZulu version was translated from English and then back translated by a second translator to ensure that the interpretation was correct. The questionnaire covered the following topics: (i) demographic information (age, marital status, employment, number of people living at home, geographic position, water source, fuel source, type of housing, housing materials, and education); (ii) physical activity during pregnancy (sitting, walking, moderate, and vigorous exercise); (iii) tobacco use during pregnancy; (iv) drug abuse during pregnancy; (v) alcohol consumption during pregnancy; and (vi) dietary intake during pregnancy assessed using a validated 48‐item unquantified food frequency questionnaire as well as a food group‐specific questionnaire about foods that contain saturated fat intake, salt intake, and sugar intake.^12^

### Medical information

Medical data were also collected from the participant's medical records including blood results, medication, and prevalence of co‐morbidities, such as GDM, HPT disorders, and anemia.

#### Gestational diabetes mellitus

GDM is defined as any degree of glucose intolerance with onset or first diagnosis during pregnancy.^13^ The participants were categorized as having GDM based on whether they were diagnosed during their pregnancy.

#### Hypertensive disorders

Hypertension is defined as having a continuous systolic blood pressure of ≥140 mmHg or diastolic blood pressure of ≥90 mmHg.^14^ The participants were categorized as having HPT with the presence of either: (i) chronic hypertension defined as hypertension predating pregnancy or diagnosed before 20 weeks of gestational age during pregnancy;^15^ (ii) pregnancy‐induced hypertension (PIH) defined as high blood pressure that occurs at ≥20 weeks of gestational age during pregnancy in a previously normotensive woman;^12^, ^14^, ^15^ (iii) preeclampsia toxemia (PET) defined as blood pressure of ≥140/90 mmHg accompanied by proteinuria or evidence of organ dysfunction at ≥20 weeks of gestational age during pregnancy;^15^ (iv) late‐onset PET defined as PET diagnosed ≥34 weeks of gestational age during pregnancy;^16^ and (v) PET characterized by the presence of hemolysis, elevated liver enzymes, and low platelet counts (HELLP).^17^

#### Anemia

Pregnancy‐associated anemia is caused by a physiological change in the vascular system whereby hemodilution occurs with an increase in intravascular blood volume without an equivalent increase in red blood cells.^18^ The hemoglobin (Hb) blood count (g/dl) was measured on admission to the hospital. Anemia was defined as having a Hb (g/dl) value of less than 11 g/dl.^19^

#### Cesarean section

A cesarean section (CS) is a surgical delivery method, whereby the baby is removed from the mother's womb by making a surgical incision in the abdomen and uterus.^20^ This procedure is indicated when natural vaginal delivery is not feasible.^20^

#### Preterm delivery

A normal human pregnancy lasts 40 weeks, and a preterm birth is defined as a baby delivered before 37 weeks of gestation.^21^

#### Geriatric pregnancy

A geriatric pregnancy is defined as a pregnancy in women ≥35 years of age.^22^

### Statistical analysis

To determine the factors associated with maternal overweight and obesity and its outcomes the BMI classification was used where overweight/obese pregnant women were classified as having a BMI of ≥25 kg/m^2^ and nonoverweight/nonobese pregnant women having a BMI of <25 kg/m^2^.^11^ Data entry and statistical analysis were performed using the statistical software packages, GraphPad Prism 5, and IBM SPSS for Windows version 27. Descriptive statistics were performed for demographic, clinical, and laboratory data. Descriptive statistics with mean (x¯) and SD, frequency, and percentages were calculated. The differences between the maternal BMI categories were assessed using Fisher's exact *t*‐test (two categories) and the *χ* ^2^ test (more than two categories). Simple and multiple logistic regression analyses were used to determine factors associated with maternal overweight and obesity. Multiple logistic regression was performed using the forward selection and backward elimination method, followed by the manual retention or removal of the independent variables remaining in the model based on their clinical importance. The output between models was then compared and the best model was selected. The independent variables were HIV status, type of pregnancy, gestational age, maternal age, marital status, job status, number of people living at home, geographic position, living conditions, education, and maternal health outcomes like CS, preterm delivery, hypertensive disorders, and anemia. In this study, the dependent variable was the BMI category, and the single dichotomous outcome was coded as 0 for nonoverweight/nonobese and 1 for overweight/obese. Simple logistic regression was performed to select the variables for multiple logistic regression analysis, and only variables with a *p*‐value <0.05. The factors included in the multiple logistic regression were gestational age, maternal age, HPT disorders, and anemia. Multicollinearity between the different predictor variables was checked using the variance inflation factor (VIF). Notably, a VIF value of <5.0 indicates no multicollinearity. All possible two‐way interaction terms between significant variables were checked individually. The adjusted odds ratio was estimated with a 95% confidence interval. A *p*‐value of <0.05 was considered statistically significant.

### Ethics approval and informed consent

The study was approved by the Biomedical Research Ethics Council (BREC) of the University of KwaZulu‐Natal (UKZN) (BE269/18), KwaZulu‐Natal Department of Health (KZNDOH) (HRKM261/18), and PMMH (29/RESH/2018). All the participants in this study had provided verbal and written consent, participated voluntarily, did not receive incentives, and had the right to withdraw at any stage of the study. Anonymity was ensured by using participant codes.

## Results

### Maternal demographic characteristics

The 200 pregnant women were categorized into overweight/obese (*n* = 97) and nonoverweight/nonobese (*n* = 103), where 45.0% (*n* = 90) were HIV‐infected and 55.0% (*n* = 110) were HIV‐uninfected. The demographic characteristics for the both HIV‐infected and HIV‐uninfected pregnant black South African women are represented in Table 1, which includes the following variables: (i) HIV status; (ii) type of pregnancy; (iii) gestational age; (iv) maternal age; (v) marital status; (vi) job status; (vii) number of people living at home; (viii) geographic position; (ix) living conditions; and (x) education. Our study findings identified that study participants (*N* = 200) were experiencing various social challenges including being single (without the support of a partner) (88.0%), being unemployed (82.5%), living in informal housing (66.5%), having a large household size (x¯ ± SD = 6.6 ± 3.9), having a lack of access to safe running water (5.0%), no access to electricity (11%), and a lack of education with 50% of the pregnant women not having achieved matriculation. Notably, the demographic characteristics were not significantly different between the pregnant women with a BMI of ≥25.0 kg/m^2^ compared to the pregnant women with a BMI of <25 kg/m^2^. However, the overweight/obese pregnant women were significantly older (x¯ ± SD = 27.9 ± 5.6) than the pregnant women with a BMI of <25 kg/m^2^ (x¯ ± SD = 26.1 ± 5.5), where 18% of the pregnant women were categorized as having a geriatric pregnancy.

**TABLE 1:** Demographic characteristics for both human immunodeficiency virus (HIV)‐infected and HIV‐uninfected pregnant black South African women categorized according to the maternal body mass index (BMI) (kg/m^2^)

Variables	All pregnant females (*n* = 200)	Maternal BMI (kg/m^2^)		*p*‐Value
		≥25.0 (*n* = 97)	<25.0 (*n* = 103)	
HIV status				
HIV infected^a^ (%)	90 (45.0)	42 (43.3)	48 (46.6)	0.6713^b^
HIV uninfected^c^ (%)	110 (55.0)	55 (56.7)	55 (53.4)	
Type of pregnancy				
Single (%)	196 (98.0)	95 (97.9)	101 (98.1)	1.000^b^
Twin (%)	4 (2.0)	2 (2.1)	2 (1.9)	1.000^b^
Gestational age (weeks)				
Mean (SD)	37.7 (2.8)	38.1 (2.4)	37.2 (3.1)	0.0982^d^
Age groups				
Mean age in years (SD)	27.0 (5.6)	27.9 (5.6)	26.1 (5.5)	0.0173^d^ ^,^ *
18–35 years (%)	178 (89.0)	84 (86.6))	94 (91.3)	0.3674^b^
> 35 years (%)	18 (11.0)	13 (13.4)	9 (8.7)	
Marital status				
Single (%)	176 (88.0)	82 (84.5)	94 (91.3)	0.1915^b^
Married (%)	10 (5.0)	8 (8.2)	2 (1.9)	0.0528^b^
Divorced (%)	0 (0.0)	0 (0.0)	0 (0.0)	—
Engaged (%)	14 (7.0)	7 (7.2)	7 (6.8)	1.0000^b^
Job‐status				
Employed (%)	35 (17.5)	18 (18.6)	17 (15.5)	0.7141^b^
Unemployed (%)	165 (82.5)	79 (81.4)	86 (83.5)	
Number of people living at home				
Mean total (SD)	6.6 (3.9)	6.1 (3.3)	7.1 (4.3)	0.0741^d^
Mean adult (SD)	3.9 (2.3)	3.6 (1.9)	4.2 (2.6)	0.0666^d^
Mean children (SD)	2.7 (2.5)	2.5 (2.1)	3.0 (2.9)	0.4947^d^
Geographic position				
Rural (%)	100 (50.0)	51 (52.6)	49 (47.5)	0.5715^b^
Urban (%)	100 (50.0)	46 (47.4)	54 (52.4)	
Living conditions				
Water source				
Inside tap (%)	93 (46.5)	43 (44.3)	50 (48.5)	0.5730^b^
Outside tap (%)	86 (43.0)	44 (45.4)	42 (40.8)	0.5684^b^
Pump (%)	11 (5.5)	6 (6.2)	5 (4.9)	0.7610^b^
River (%)	10 (5.0)	4 (4.1)	6 (5.8)	0.7487^b^
Housing material used to make a home				
Plastic/cardboard (%)	4 (2.0)	1 (1.0)	3 (2.9)	0.6219^b^
Mud (%)	6 (3.0)	2 (2.1)	4 (3.9)	0.6836^b^
Mud and cement (%)	48 (24.0)	27 (27.8)	21 (20.4)	0.2481^b^
Corrugated iron/zinc (%)	118 (59.0)	61 (62.9)	57 (55.3)	0.3151^b^
Bare brick/cement block (%)	107 (53.5)	50 (51.5)	57 (55.3)	0.6707^b^
Plaster/finished (%)	57 (28.5)	26 (26.8)	31 (30.1)	0.6406^b^
Housing type				
Formal (%)	67 (33.5)	34 (35.1)	33 (32.0)	0.6566^b^
Informal (%)	133 (66.5)	63 (64.9)	70 (68.0)	
Fuel source				
Electricity (%)	178 (89.0)	88 (90.7)	90 (87.4)	0.5034^b^
Paraffin (%)	22 (11.0)	8 (8.2)	14 (13.6)	0.2635^b^
Gas (%)	21 (10.5)	6 (6.2)	15 (14.6)	0.0657^b^
Wood (%)	64 (32.0)	28 (28.9)	36 (35.0)	0.3677^b^
Education				
≤ Grade 7 (%)	5 (2.5)	2 (1.0)	3 (1.5)	1.0000^b^
Grade 8–11 (%)	95 (47.5)	43 (21.5)	52 (26.0)	0.3988^b^
Grade 12 (%)	56 (28.0)	26 (26.8)	30 (29.1)	0.7543^b^
Tertiary studies‐incomplete (%)	29 (14.5)	17 (17.5)	12 (11.7)	0.3153^b^
Tertiary studies‐diploma (%)	13 (6.5)	7 (7.2)	6 (5.8)	0.7783^b^
Tertiary studies‐degree (%)	2 (1.0)	2 (2.1)	0 (0)	0.2340^b^

^a^ Pregnant women have been tested for HIV infection and results are positive, they are receiving antiretroviral treatment.

^b^ Fisher's exact test.

^c^ Pregnant women have been test for the HIV infection and results are negative.

^d^ Mann–Whitney *t*‐test.

* Results are statistically significant *p* < 0.05.

### Maternal physical activity and lifestyle characteristics

The maternal physical activity and lifestyle characteristics in both HIV‐infected and HIV‐uninfected pregnant black South African women are represented in Table 2, which includes the following variables: (i) physical activity; (ii) vigorous physical activity; (iii) moderate physical activity; (iv) travel by walking; (v) smoking; (vi) drugs; and (vii) alcohol consumption during pregnancy. This study identified that 20.5% of the pregnant women (*N* = 200) engaged in physical exercise during their pregnancy, which is one in five pregnant women. Our study findings investigated whether the pregnant women engaged in risky behavior during pregnancy such as alcohol consumption, drug abuse, and smoking during pregnancy. It was identified that 24.0% of the pregnant women (*N* = 200) consumed alcohol during pregnancy, which is one in four pregnant women. Only 1.0% of the pregnant women (*N* = 200) participated in drug abuse during pregnancy. Two percent (2%) of the pregnant women (*N* = 200) chose to smoke during their pregnancy. Whereas 29.0% of the pregnant women (*N* = 200) were unwillingly exposed to second‐hand smoke during their pregnancy, which is 1 in 3 pregnant women. Notably, the physical activity and lifestyle characteristics were not significantly different between the pregnant women with a BMI of ≥25.0 kg/m^2^ compared to the pregnant women with a BMI of <25 kg/m^2^.

**TABLE 2:** Physical activity and lifestyle characteristics in both human immunodeficiency virus (HIV)‐infected and HIV‐uninfected pregnant black South African women categorized according to the maternal body mass index (BMI) (kg/m^2^)

Variables	All pregnant females (*n* = 200)	Maternal BMI (kg/m^2^)		*p*‐Value
		<25.0	≥25.0	
Physical activity	(*n* = 200)	(*n* = 103)	(*n* = 97)	0.2201^a^
Exercise during pregnancy (%)	41 (20.5)	25 (24.3)	16 (16.5)	
Vigorous activity (%)	11 (6.0)	9 (56.3)	2 (12.5)	0.0595^a^
Mean number of days per week (SD)	4.0 (2.5)	4.4 (2.3)	2.0 (1.0)	—
Mean min per day (SD)	51.0 (36.9)	54 (36.8)	37.5 (22.5)	—
Moderate activity (%)	34 (17.0)	19 (76.0)	15 (93.8)	0.7069^a^
Mean number of days per week (SD)	3.1 (1.8)	3.3 (1.9)	2.9 (1.5)	0.8027^b^
Mean min per day (SD)	38.0 (35.9)	35.0 (32.5)	44.3 (39.6)	0.2260^b^
The mean number of hours spent sitting in the previous week before admission (SD)	25.8 (23.5)	4.7 (2.8)	5.4 (3.0)	0.2542^b^
Travel by walking (%)	175 (87.5)	90 (87.4)	85 (87.6)	1.000^a^
Mean number of days per week (SD)		3.0 (1.9)	3.0 (2.0)	0.8650^b^
Mean min per day (SD)		28.8 (23.6)	30.7 (22.0)	0.1899^b^
Smoking/ exposure	(*n* = 200)	(*n* = 103)	(*n* = 97)	0.3567^a^
Currently smoking (%)	4 (2.0)	1 (1.0)	3 (3.1)	
Smoked before pregnancy (%)	11 (6.0)	6 (5.8)	5 (5.2)	1.000^a^
Exposure to in‐house second‐hand smoking (%)	58 (29.0)	34 (33.0)	24 (24.7)	0.2152^a^
Exposure to second‐hand smoking at work (%)	6 (3.0)	4 (23.5)	2 (11.1)	0.4018^a^
Exposure to industrial smoke at work (%)	4 (2.0)	2 (11.8)	2 (11.1)	1.0000^a^
Drugs (%)	2 (1.0)	1 (0.5)	1 (0.5)	1.0000^a^
Alcohol intake during pregnancy (%)	48 (24.0)	27 (13.5)	21 (10.5)	0.5091^a^

^a^ Fisher's exact test.

^b^ Mann–Whitney *t*‐test.

### Maternal food frequency intake during pregnancy

This study investigated the retrospective food frequency intake of obesity in both HIV‐infected and HIV‐uninfected pregnant black South African women during their pregnancy. The food groups that were assessed were: (i) saturated fat; (ii) cooking fat; (iii) salt; (iv) sugar; (v) animal protein; (vi) dairy; (vii) legume; (viii) starch; (ix) vegetables; and (x) fruit (see Table 3). Overall, the dietary pattern identified for the overweight and obese pregnant women in comparison to the nonoverweight/nonobese pregnant women was that their diet was higher in saturated fat, higher in salt, higher in sugar, higher in animal protein, lower in dairy, higher in legumes, higher in starch, higher in vegetables, and had a similar intake of fruit. However, these dietary patterns were not significantly different between the pregnant women with a BMI of ≥25.0 kg/m^2^ compared to the pregnant women with a BMI of <25 kg/m^2^.

**TABLE 3:** Maternal food frequency intake in both human immunodeficiency virus (HIV)‐infected and HIV‐uninfected pregnant black South African women divided into high, moderate, and low, and further categorized according to the maternal body mass index (BMI) (kg/m^2^)

Food group	Maternal BMI (kg/m^2^)		*p*‐Value^a^
	<25.0	≥25.0	
Saturated fat intake	(*n* = 97)	(*n* = 97)	0.616
Low (%)	2 (2.1)	1 (1.0)	
Moderate (%)	48 (49.5)	43 (44.3)	
High (%)	47 (48.5)	53 (54.6)	
Cooking fat intake	(*n* = 93)	(*n* = 92)	0.608
Low (%)	1 (1.1)	0 (0.0)	
Moderate (%)	57 (61.3)	57 (62.0)	
High (%)	35 (37.6)	35 (38.0)	
Salt intake^b^	(*n* = 96)	(*n* = 96)	0.275
Low (%)	6 (6.3)	3 (3.1)	
Moderate (%)	50 (52.1)	60 (62.5)	
High (%)	40 (41.7)	33 (34.4)	
Sugar intake	(*n* = 97)	(*n* = 97)	0.284
Low (%)	11 (11.3)	5 (5.2)	
Moderate (%)	21 (21.6)	21 (21.6)	
High (%)	65 (67.0)	77 (79.4)	
Animal protein intake	(*n* = 94)	(*n* = 93)	0.424
Low (%)	29 (30.9)	22 (23.7)	
Moderate (%)	56 (59.6)	58 (62.4)	
High (%)	9 (9.6)	13 (14.0)	
Dairy intake	(*n* = 94)	(*n* = 93)	0.872
Low (%)	35 (37.2)	38 (40.9)	
Moderate (%)	35 (37.2)	32 (34.4)	
High (%)	24 (25.5)	23 (24.7)	
Legume intake	(*n* = 94)	(*n* = 92)	0.138
Low (%)	64 (68.1)	50 (54.3)	
Moderate (%)	22 (23.4)	33 (35.9)	
High (%)	8 (8.5)	9 (9.8)	
Starch intake	(*n* = 94)	(*n* = 92)	0.210
Low (%)	12 (12.8)	9 (9.8)	
Moderate (%)	43 (45.7)	54 (58.7)	
High (%)	39 (41.5)	29 (31.5)	
Vegetable intake	(*n* = 93)	(*n* = 91)	0.185
Low (%)	11 (11.8)	7 (7.7)	
Moderate (%)	50 (53.8)	41 (45.0)	
High (%)	32 (34.4)	43 (47.3)	
Fruit intake	(*n* = 95)	(*n* = 91)	0.963
Low (%)	27 (28.4)	27 (29.7)	
Moderate (%)	50 (52.6)	48 (52.7)	
High (%)	18 (18.9)	16 (17.6)	

^a^ Pearson chi‐square test.

^b^ Salt intake from food.

### Factors associated with maternal overweight and obesity

A simple logistic regression showed that maternal age and gestational age were significantly associated with maternal overweight and obesity in both HIV‐infected and HIV‐uninfected pregnant black South African women (see Table 4). Notably, there were no significant associations between HIV status, type of pregnancy, marital status, job status, number of people living at home, geographic position, water source, housing type, fuel source, education, physical activity, smoking, alcohol, and drugs with maternal overweight and obesity. Multiple logistic regression analysis showed that maternal age and gestational age were significantly associated with maternal overweight and obesity in both HIV‐infected and HIV‐uninfected pregnant women (see Table 5). Hence, for every 1‐year unit increase in the age of the participants, they were 1.061 times more likely to be overweight/obese. This suggests that as the maternal age increases, so does the weight of the mother. Likewise with gestational age, for every 1‐week increase, the participants were 1.121 times more likely to be overweight/obese. This suggests that as the gestational age of the pregnancy increases, so does the weight of the mother.

**TABLE 4:** Simple logistic regression of factors associated with maternal overweight and obesity (*n* = 200) obesity in both HIV‐infected and HIV‐uninfected pregnant black South African women

Variables	Regression coefficient B	Crude OR (95% CI)	Wald statistic (df)	*p*‐Value
HIV status				
Infected^a^		1		
Uninfected^b^	0.134	1.143 (0.654–1.996)	0.220 (1)	0.639
Type of pregnancy				
Single	−0.61	0.941 (0.130–6.812)	0.004 (1)	0.952
Twin		1		
Gestational age	0.114	1.121 (1.006–1.248)	4.284 (1)	0.038*
Maternal age	0.060	1.061 (1.009–1.117	5.274 (1)	0.022*
Geriatric maternal age^c^	−0.480	0.619 (0.252–1.521)	1.095 (1)	0.295
Marital status				
Single	−0.137	0.872 (0.294–2.591)	0.060 (1)	0.806
Married	1.386	4.000 (0.616–25.964)	2.110 (1)	0.146
Engaged		1		
Job‐status				
Employed		1		
Unemployed	−0.142	0.868 (0.418–1.800)	0.146 (1)	0.703
Number of people living at home				
Total	−0.073	0.930 (0.861–1.005)	3.353 (1)	0.067
Adults	−0.130	0.878 (0.770–1.001)	3.812 (1)	0.051
Children	−0.073	0.930 (0.830–1.041)	1.582 (1)	0.208
Geographic position				
Rural		1		
Urban	−0.200	0.818 (0.470–1.426)	0.500 (1)	0.479
Water source				
Inside tap	0.255	1.290 (0.341–4.874)	0.141 (1)	0.707
Outside tap	0.452	1.571 (0.414–5.965)	0.441 (1)	0.507
Pump	0.588	1.800 (0.318–10.201)	0.441 (1)	0.507
River		1		
Housing type				
Formal		1		
Informal	−0.135	1.145 (0.636–2.060)	0.203 (1)	0.652
Fuel source				
Electricity	−0.428	0.652 (0.178–2.389)	0.417 (1)	0.518
Paraffin	−1.099	0.333 (0.051–2.177)	1.317 (1)	0.251
Gas	−21.608	0.000 (0.000–0.000)	0.000 (1)	0.999
Wood		1		
Education				
≤Grade 7	−21.608	0.000 (0.000–0.000)	0.000 (1)	0.999
Grades 8–11	−21.393	0.000 (0.000–0.000)	0.000 (1)	0.999
Grade 12	−21.346	0.000 (0.000–0.000)	0.000 (1)	0.999
Tertiary studies: incomplete	−20.855	0.000 (0.000–0.000)	0.000 (1)	0.999
Tertiary studies: diploma	−21.049	0.000 (0.000–0.000)	0.000 (1)	0.999
Tertiary studies: degree		1		
Physical activity				
Yes		1		
No	0.484	1.623 (0.806–3.268)	1.835 (1)	0.176
Vigorous activity				
Yes	−1.371	0.254 (0.047–1.379)	2.521 (1)	0.112
No		1		
Moderate activity				
Yes	0.862	2.368 (0.417–13.461)	0.946 (1)	0.331
No		1		
Travel by walking				
Yes		1		
No	−0.023	0.977 (0.422–2.261)	0.003 (1)	0.957
Currently smoking				
Yes		1		
No	−1.180	0.307 (0.031–3.005)	1.029 (1)	0.310
Smoked before pregnancy				
Yes		1		
No	0.129	1.138 (0.336–3.857)	0.043 (1)	0.835
Second‐hand smoking (house)				
Yes		1		
No	0.405	1.499 (0.808–2.779)	1.650 (1)	0.199
Second‐hand smoking (work)				
Yes		1		
No	0.652	1.919 (0.343–10.724)	0.551 (1)	0.458
Industrial smoke (work)				
Yes	−0.61	1	0.004 (1)	0.952
No		0.941 (0.130–6.812)		
Drugs				
Yes		1		
No	−0.061	0.941 (0.058–15.259)	0.002 (1)	0.966
Alcohol				
Yes		1		
No	0.251	1.286 (0.669–2.470)	0.569 (1)	0.451
Saturated fat intake				
Low		1		
Moderate	0.583	2.255 (0.198–25.679)	0.220 (1)	0.639
High	0.813	1.792 (0.157–20.463)	0.429 (1)	0.512
Cooking fat intake				
Low		1		
Moderate	21.203	16.5 (0.000–0.000)	0.000 (1)	1.000
High	21.203	16.5 (0.000–0.000)	0.000 (1)	1.000
Salt intake				
Low		1		
Moderate	0.875	2.400 (0.571–10.087)	1.428 (1)	0.232
High	0.501	1.650 (0.383–7.109)	0.452 (1)	0.502
Sugar intake				
Low		1		
Moderate	0.788	2.200 (0.651–7.436)	1.610 (1)	0.205
High	0.877	2.403 (0.792–7.287)	2.399 (1)	0.121
Animal protein intake				
Low		1		
Moderate	0.311	1.365 (0.702–2.654)	0.843 (1)	0.359
High	0.644	1.904 (0.690–5.252)	1.548 (1)	0.213
Dairy intake				
Low		1		
Moderate	−0.172	0.842 (0.434–1.636)	0.257 (1)	0.612
High	−0.125	0.883 (0.424–1.838)	0.111 (1)	0.739
Legume intake				
Low		1		
Moderate	0.652	1.920 (0.998–3.693)	3.820 (1)	0.051
High	0.365	1.440 (0.518–4.000)	0.489 (1)	0.484
Starch intake				
Low		1		
Moderate	0.515	1.674 (0.646–4.341)	1.125 (1)	0.289
High	−0.009	0.991 (0.369–2.665)	0.000 (1)	0.986
Vegetable intake				
Low		1		
Moderate	0.254	1.289 (0.458–3.623)	0.231 (1)	0.631
High	0.747	2.112 (0.737–6.048)	1.938 (1)	0.164
Fruit intake				
Low		1		
Moderate	−0.062	0.940 (0.483–1.829)	0.033 (1)	0.855
High	−0.118	0.889 (0.376–2.099)	0.072 (1)	0.788

Abbreviations: CI, confidence interval; HIV, human immunodeficiency virus; OR, odds ratio.

^a^ Pregnant women have been tested for HIV infection and results are positive, they are receiving antiretroviral treatment.

^b^ Pregnant women have been tested for the HIV infection and results are negative.

^c^ ≥35 years.

* Results are statistically significant *p* < 0.05.

**TABLE 5:** Multiple logistic regression of factors associated with maternal overweight and obesity (*n* = 200) in both HIV‐infected and HIV‐uninfected pregnant women

Variables	Regression coefficient B	Crude OR^a^ (95% CI)	Wald statistic (df)	*p*‐Value
Gestational age	0.114	1.121 (1.005–1.251)	4.183 (1)	0.041*
Maternal age	0.059	1.061 (1.008–1.117)	5.189 (1)	0.023*

Abbreviations: CI, confidence interval; HIV, human immunodeficiency virus; OR, odds ratio.

^a^ Model has been adjusted for gestational age and maternal age.

* Results are statistically significant *p* < 0.05.

### Association between maternal overweight/obesity and maternal health outcomes

**FIGURE 2:**
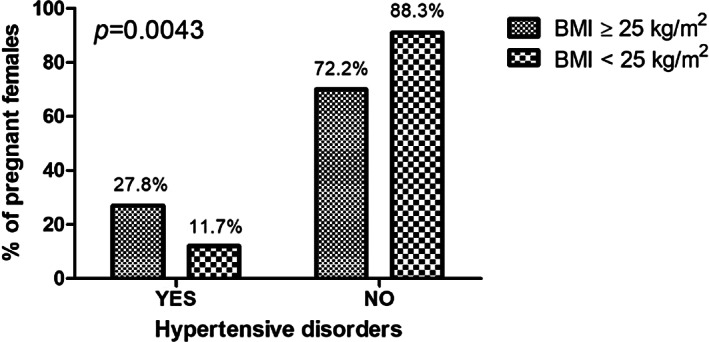
Fisher's exact *t*‐test showing the prevalence of hypertensive disorders in both human immunodeficiency virus (HIV)‐infected and HIV‐uninfected pregnant black South African women categorized according to body mass index (BMI) (kg/m^2^).

**FIGURE 3:**
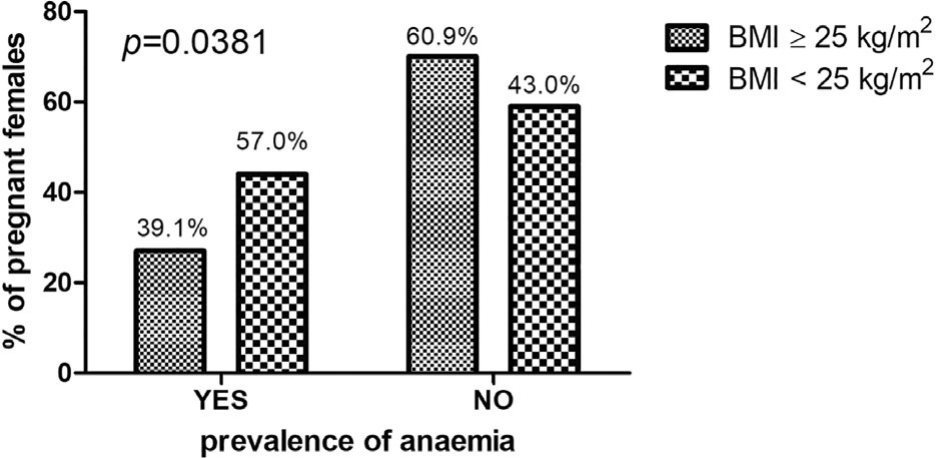
Fisher's exact *t*‐test showing the prevalence of anemia in both human immunodeficiency virus (HIV)‐infected and HIV‐uninfected pregnant black South African women categorized according to maternal body mass index (BMI) (kg/m^2^).

The associations between maternal health outcomes and maternal overweight and obesity in both HIV‐infected and HIV‐uninfected pregnant black South African women are summarized in Table 6. In our study, 39.5% of the pregnant women had a CS, 24.0% had a preterm birth, and a small percentage of 1.5% had GDM. Notably, CS, preterm birth, GDM, PIH, PET, PET, and HELLP and late onset PET were not significantly different between the pregnant women with a BMI of ≥25.0 kg/m^2^ compared to the pregnant women with a BMI of <25 kg/m^2^. However, HPT disorders (*p* = 0.0043), chronic HPT (*p* = 0.0003), mean Hb (g/dl) (*p* = 0.0409), and anemia (*p* = 0.0381) were significantly different between the pregnant women with a BMI of ≥25.0 kg/m^2^ compared to the pregnant women with a BMI of <25 kg/m^2^. The prevalence of hypertensive disorders in the pregnant women (*N* = 200) was 19.5%, with the highest prevalence being in the overweight and obese group of pregnant women (*n* = 27; 27.8%) (see Figure 2). These hypertensive disorders included chronic HPT (*n* = 11; 5.5%), PET (*n* = 15; 7.5%), and PIH (*n* = 13; 6.5%). In the present study, 48.6% of the pregnant women had anemia, with the highest prevalence in pregnant women with a BMI <25 kg/m^2^ (*n* = 44; 57.0%) than compared to overweight and obese pregnant women (*n* = 27; 39.1%) (see Figure 3). A simple logistic regression showed that hypertensive disorders, anemia, and Hb (g/dl) were significantly associated with maternal overweight and obesity in both HIV‐infected and HIV‐uninfected pregnant black South African women (see Table 7). Notably, there were no significant associations with CS, preterm delivery, GDM, and PET. A simple logistic regression showed that hypertensive disorders were significantly associated with maternal overweight and obesity in HIV‐infected pregnant black South African women (see Table 8). Notably, there were no significant associations with CS, preterm delivery, GDM, PET, and anemia. Multiple logistic regression analysis showed that hypertensive disorders and anemia were significantly associated with maternal overweight and obesity in both HIV‐infected and HIV‐uninfected pregnant black South African women (see Table 9). Hence, overweight and obese pregnant women were 2.420 times more likely to not have anemia in comparison to those with a BMI <25 kg/m^2^. However, overweight and obese pregnant women were 0.273 times more likely to have hypertensive disorders in comparison to those with a BMI <25 kg/m^2^. Therefore, maternal overweight and obesity increase the risk for hypertensive disorders during pregnancy but decreases the risk for anemia during pregnancy.

**TABLE 6:** Maternal health outcomes in both HIV‐infected and HIV‐uninfected pregnant black South African women categorized according to the maternal BMI (kg/m^2^)

Variables	All pregnant women (*N* = 200)	Maternal BMI (kg/m^2^)		*p*‐Value
		*n* = 103	*n* = 97	
		<25.0	≥25.0	
Cesarean section (%)	79 (39.5)	37 (35.9)	42 (43.3)	0.1783^a^
Preterm delivery (%)	48 (24.0)	30 (29.1)	18 (18.6)	0.0562^a^
GDM (%)	3 (1.5)	1 (1.0)	2 (2.1)	0.6120^a^
Hypertensive disorders (%)	39 (19.5)	12 (11.7)	27 (27.8)	0.0043^a^ ^,^ *
Chronic HPT (%)	11 (5.5)	0 (0.0)	11 (11.3)	0.0003^a^ ^,^ *
PIH (%)	13 (6.5)	6 (5.8)	7 (7.2)	0.7783^a^
PET (%)	15 (7.5)	6 (5.8)	9 (9.3)	0.4260^a^
HELLP and PET (%)	1 (0.5)	1 (16.7)	0 (0.0)	1.0000^a^
Late onset PET (%)	2 (1.0)	1 (16.7)	1 (11.1)	1.0000^a^
Anemia	(*n* = 146)	(*n* = 77)	(*n* = 69)	
Mean Hb in g/dl (SD)	11.0 (1.6)	10.7 (1.6)	11.2 (1.6)	0.0409^b^ ^,^ *
Prevalence of anemia (%)	71 (48.6)	44 (57.0)	27 (39.1)	0.0381^a^ ^,^ *

Abbreviations: BMI, body mass index; GDM, gestational diabetes mellitus; Hb, hemoglobin; HIV, human immunodeficiency virus; HPT, hypertension; PET, preeclampsia toxemiatoxaemia; PIH, pregnancy‐induced hypertension.

^a^ Fisher's exact test.

^b^ Mann–Whitney *t*‐test.

* Results are statistically significant *p* < 0.05.

**TABLE 7:** Simple logistic regression of maternal health outcomes associated with maternal overweight and obesity in both HIV‐infected and HIV‐uninfected pregnant black South African women (*N* = 200)

Variables	Regression coefficient B	Crude OR (95% CI)	Wald statistic (df)	*p*‐Value
Cesarean section				
Yes		1		
No	−0.309	0.734 (0.416–1.296)	1.135 (1)	0.287
Preterm delivery				
Yes		1		
No	0.590	1.804 (0.927–3.508)	3.019 (1)	0.082
GDM				
Yes		1		
No	−0.764	0.466 (0.042–5.220)	0.384 (1)	0.535
Hypertensive disorder				
Yes		1		
No	−1.073	0.342 (0.162–0.722)	7.909 (1)	0.005*
PET				
Yes		1		
No	−0.503	0.605 (0.207–1.768)	0.844 (1)	0.358
Anemia				
Yes		1		
No	0.659	1.933 (1.070–3.492)	4.777 (1)	0.029*
Hb (g/dl)	0.211	1.235 (1.003–1.520)	3.961 (1)	0.047*

Abbreviations: GDM, gestational diabetes mellitus; Hb, hemoglobin; HPT, hypertension; PET, preeclampsia toxemia; PIH, pregnancy‐induced hypertension.

* Results are statistically significant *p* < 0.05.

**TABLE 8:** Simple logistic regression of maternal health outcomes associated with maternal overweight and obesity pregnant black South African women, adjusted for HIV status

Variables	Regression coefficient B	Crude OR (95% CI)	Wald statistic (df)	*p*‐Value
Cesarean section				
HIV‐infected				
Yes		1		
No	−0.402	0.669 (0.277–1.614)	0.800 (1)	0.371
HIV‐uninfected				
Yes		1		
No	−0.221	0.802 (0.377–1.703)	0.331 (1)	0.565
Preterm delivery				
HIV‐infected				
Yes		1		
No	0.567	1.318 (0.513–3.387)	0.328 (1)	0.567
HIV‐uninfected				
Yes				
No	0.880	2.410 (0.933–6.226)	3.302 (1)	0.069
GDM				
HIV‐infected				
Yes		1		
No	21.090	144 (0.000–0.000)	0.000 (1)	1.000
HIV‐uninfected				
Yes		1		
No	−21.240	0.0 (0.000–0.000)	0.000 (1)	0.999
Hypertensive disorder				
HIV‐infected				
Yes		1		
No	−1.459	0.233 (0.075–0.717)	6.439 (1)	0.011*
HIV‐uninfected				
Yes		1		
No	−0.753	0.471 (0.172–1.291)	2.142 (1)	0.143
PET				
HIV‐infected				
Yes		1		
No	−0.150	0.860 (0.231–3.206)	0.050 (1)	0.860
HIV‐uninfected				
Yes		1		
No	−1.443	0.236 (0.026–2.184)	1.618 (1)	0.203
Anemia				
HIV‐infected				
Yes		1		
No	0.504	1.656 (0.709–3.867)	1.359 (1)	0.244
Hb (g/dl)	0.170	1.185 (0.869–1.618)	1.149 (1)	0.284
HIV‐uninfected				
Yes		1		
No	0.794	2.213 (0.956–5.126)	3.437 (1)	0.064
Hb (g/dl)	0.245	1.278 (0.957–1.705)	2.767 (1)	0.096

Abbreviations: CI, confidence interval; GDM, gestational diabetes mellitus; Hb, hemoglobin; HPT, hypertension; OR, odds ratio; PET, preeclampsia toxemia; PIH, pregnancy‐induced hypertension.

* Results are statistically significant *p* < 0.05.

**TABLE 9:** Multiple logistic regression of maternal health outcomes associated with maternal overweight and obesity in both HIV‐infected and HIV‐uninfected pregnant black South African women (*n* = 200)

Variables	Regression coefficient B	Crude OR^a^ (95% CI)	Wald statistic (df)	*p*‐Value
Hypertensive disorder				
Yes		1		
No	−1.300	0.273 (0.124–0.601)	10.401 (1)	0.001*
Anemia				
Yes		1		
No	0.884	2.420 (1.283–4.563)	7.454 (1)	0.006*

Abbreviations: CI, confidence interval; OR, odds ratio.

^a^ Model has been adjusted for Hypertensive disorders and anemia.

* Results are statistically significant *p* < 0.05.

## Discussion

In our cross‐sectional study of both HIV‐infected and HIV‐uninfected pregnant black South African women, we identified that maternal overweight and obesity in pregnancy were significantly associated with maternal age, gestational age, HPT disorders, and anemia. In a setting where there is both a high prevalence of overweight and obesity in women of child‐bearing age and a high prevalence of HIV in pregnant women, our findings highlight the need for weight management interventions during pregnancy to minimize the adverse maternal health outcomes.^23^, ^24^

In the present study, geriatric pregnancy was not associated with maternal overweight and obesity, but it was identified that maternal age was associated with maternal overweight and obesity in both HIV‐infected and HIV‐uninfected pregnant black South African women. Similar findings were found in an Australian study, which identified that increasing maternal BMI was associated with increasing maternal age (*p* < 0.001) and suggested that older women were at a higher risk of becoming overweight and obese in pregnancy.^25^ In a Lithuanian study, obese pregnant women were also significantly older than the normal‐weight pregnant women (*p* < 0.001).^26^ Similarly other African studies have identified that the risk for overweight/obesity was higher among older women.^27^, ^28^, ^29^, ^30^ The mechanisms responsible for this age‐associated risk with gestational weight gain may be linked to metabolic dysfunction and alterations in the deposition of adipose tissue.^31^

Gestational age in this study was also positively associated with overweight and obesity in both HIV‐infected and HIV‐uninfected pregnant black South African women. Typically, pregnant women are encouraged to gain weight as their gestational age progresses, with the goal of the total amount of weight gain for the 40 weeks of gestation based on the mother's prepregnancy actual body weight (kg).^32^, ^33^ Therefore, maternal overweight and obesity in pregnancy have been attributed to excessive gestational weight gain, which is defined as maternal weight gain more than the recommended amount over the course of pregnancy.^32^, ^34^ Hence, this excessive gestational weight gain should be avoided because it has been linked with increased risks of delivery complications like CS, increased risk of postpartum weight retention for the mother, miscarriage, HPT disorders, and GDM.^32^, ^34^ Even more so, for pregnant women who are already overweight/obese at the onset of pregnancy, weight gain should be carefully monitored throughout their pregnancy.^34^

Interestingly, our study found that modifiable lifestyle factors like physical activity, dietary intake, smoking, alcohol intake, and drug abuse were all not associated with overweight and obesity in both HIV‐infected and HIV‐uninfected pregnant black South African women. In our study findings, the food frequency intake was not associated with overweight and obesity in pregnancy. But the dietary patterns identified were consistent with other studies that have linked similar dietary patterns to obesity in pregnancy including a high intake of energy‐dense foods, especially foods high in saturated fat, salt, and sugar.^35^, ^36^ We found that alcohol consumption was not associated with overweight and obesity in pregnancy, but other studies have linked alcohol consumption to weight gain due to its energy density and effects on fatty acid metabolism.^37^, ^38^ Also, we found that smoking was not significantly associated with overweight and obesity in pregnancy. However, smoking during pregnancy has been associated with increased risk of excessive gestational weight gain and should be avoided.^39^ For physical activity, as pregnant women progress through their pregnancy it is common to find that their activity level decreases.^40^, ^41^ For women living in Africa, there are various barriers that prevent them from engaging in physical activity such as lack of time, lack of knowledge, inadequate information from healthcare providers, feelings of tiredness, and a lack of social support.^41^ But physical activity or exercise should be encouraged during pregnancy because it has been associated with a reduction in gestational weight gain and inversely associated with adverse maternal health outcomes like HD and GDM.^40^, ^42^

Hypertension is considered a preventable complication of pregnancy, but it is a life‐threatening condition when untreated or mismanaged.^14^, ^43^ In South Africa, HPT is considered the most direct cause of maternal mortality and accounts for 18% of maternal deaths.^43^ The mechanisms involved in the pathogenesis of obesity‐related HPT in pregnancy are caused by physiological changes in adiposity, increased blood circulation, sympathetic nervous system overactivation, stimulation of the renin‐angiotensin‐aldosterone system, alterations in adipose‐derived cytokines, insulin resistance, and structural as well as functional renal changes.^44^ Overall, we identified that HD disorders were significantly associated with maternal overweight and obesity in both HIV‐infected and HIV‐uninfected pregnant black South African women, where one in five pregnant women were affected by HPT disorders. Maternal overweight and obesity have been associated with a significantly different cardiovascular disease profile and an adverse metabolic profile compared to pregnant women with a BMI <25.0 kg/m^2^.^45^ This altered metabolic profile leads to increased risk for developing HPT disorders in pregnancy.^45^ For example, in a retrospective cohort study it was identified that maternal overweight or obesity in pregnancy was associated with a significantly increased odds of developing hypertensive disorders in comparison to pregnant women with a BMI <25 kg/m^2^.^46^ In terms of the HIV infection and treatment thereof, it is of particular interest in South African pregnant women, because it has been linked to vascular endothelial dysfunction.^47^ Our study identified that overweight and obese HIV‐infected pregnant women had increased odds for developing HPT disorders. Another study has supported this, where HIV‐infected pregnant women with a BMI ≥25 kg/m^2^ had a significantly increased odds of HPT disorders like PET (OR = 3.0; 95% CI: 1.5–6.0).^48^ Hence, this has highlighted that the HIV‐infected pregnant women who have a BMI ≥25.0 kg/m^2^ have a different cardiometabolic risk to the HIV‐uninfected pregnant women with a BMI <25 kg/m^2^.

Our study findings identified that one in three pregnant women was affected by anemia. Anemia is a common blood condition experienced by many pregnant women and is associated with an increased risk for maternal mortality in pregnancy.^19^, ^49^ Iron deficiency is one of the most common causes of anemia in South Africa, and preventative measures have been routinely implemented to prevent anemia via prophylactic iron supplementation.^49^ Despite these types of interventions already in place, the present study showed that anemia is still a cause for concern during pregnancy. However, our study identified that anemia was inversely associated with maternal overweight and obesity in both HIV‐infected and HIV‐uninfected pregnant black South African women. Our study findings are consistent with that of other studies that have investigated the association between maternal BMI and risk for anemia. In a Chinese study, where women of childbearing age with overweight (OR = 0.72; 95% CI: 0.62–0.89) and obesity (OR = 0.59; 95% CI: 0.43–0.79) were less likely to be anemic as compared to normal‐weight women.^50^ A study conducted in Ghana showed a similar pattern where differences in BMI influenced the risk for anemia, where pregnant women who were underweight had an increased odds for anemia compared to the normal‐weight pregnant women (OR = 3.17; 95% CI: 1.19–8.32).^51^ Also, in the prospective cohort study, a higher BMI in early pregnancy was associated with a higher Hb (g/dl) at the first antenatal booking and with a reduced risk of anemia in Indonesian and Ghanaian pregnant women.^52^ Overall, our study identified that the pregnant women with a BMI ≥25 kg/m^2^ were less likely to be anemic compared to those with a BMI <25 kg/m^2^. The mechanisms behind this have yet to be investigated, but it could be linked to the overweight/obese pregnant women dietary intake with possible consumption of higher quantities of bioavailable iron‐rich foods, for example, in South Africa, staple foods, such as maize and bread are fortified with iron.^53^

In conclusion, maternal overweight/obesity in both HIV‐infected and HIV‐uninfected pregnant black South African women was significantly associated with maternal age, gestational age, HPT disorders, and anemia. Maternal overweight/obesity decreased the odds for anemia but increased the odds for the development of HPT disorders, especially in the HIV‐infected pregnant women.

## Author contributions

Christen Erasmus: conception, design, data collection, statistical analysis, manuscript drafting and editing. Anil Chuturgoon and Niren Ray Maharaj: conception, design, and manuscript editing. All authors commented on previous versions of the manuscript; all authors read and approved the final manuscript.

## Conflict of Interest

The authors declare no conflict of interest.

## Data Availability

The data that support the findings of this study are available from the corresponding author upon reasonable request.
